# 

**Published:** 1949-01

**Authors:** 


### CONTENTS

| | Page |
|---|---|
| Obstetrics and Gynæcology Old and New. By H. J. DREW SMYTHE, *M.C.*, T.D. | 1 |
| The Thirty-sixth Long Fox Memorial Lecture: Some Aspects of Hospital Economy. By R. MILNES WALKER, M.S. | 7 |
| The Quadruplets. I.—DELIVERY: By H. L. SHEPHERD, Ch.M., M.B., F.R.C.O.G. AND MABEL F. POTTER, M.D., F.R.C.O.G. II.—MANAGEMENT: By BERYL D. CORNER, M.D., M.R.C.P. III.—NOTES: By E. J. WATSON-WILLIAMS, B.A. | 14 |
| Editorial Notes | 20 |
| Reviews of Books | 20 |
| Meetings of Societies | 22 |
| Local Medical Notes | 28 |

